# Brillouin Frequency Shift of Fiber Distributed Sensors Extracted from Noisy Signals by Quadratic Fitting

**DOI:** 10.3390/s18020409

**Published:** 2018-01-31

**Authors:** Hanrong Zheng, Zujie Fang, Zhaoyong Wang, Bin Lu, Yulong Cao, Qing Ye, Ronghui Qu, Haiwen Cai

**Affiliations:** 1 Shanghai Key Laboratory of All Solid-State Laser and Applied Techniques, Shanghai Institute of Optics and Fine Mechanics, Chinese Academy of Sciences, Shanghai 201800, China; hrzheng@siom.ac.cn (H.Z.); zjfang@siom.ac.cn (Z.F.); lubin@siom.ac.cn (B.L.); yeqing@siom.ac.cn (Q.Y.); rhqu@siom.ac.cn (R.Q.); 2 University of Chinese Academy of Sciences, Beijing 100049, China; 3 Nanjing Pioneer Lasersensing, Nanjing 210019, China; zerone.cyl@gmail.com

**Keywords:** fiber optics sensors, optical time domain reflectometry, scattering, Brillouin

## Abstract

It is a basic task in Brillouin distributed fiber sensors to extract the peak frequency of the scattering spectrum, since the peak frequency shift gives information on the fiber temperature and strain changes. Because of high-level noise, quadratic fitting is often used in the data processing. Formulas of the dependence of the minimum detectable Brillouin frequency shift (BFS) on the signal-to-noise ratio (SNR) and frequency step have been presented in publications, but in different expressions. A detailed deduction of new formulas of BFS variance and its average is given in this paper, showing especially their dependences on the data range used in fitting, including its length and its center respective to the real spectral peak. The theoretical analyses are experimentally verified. It is shown that the center of the data range has a direct impact on the accuracy of the extracted BFS. We propose and demonstrate an iterative fitting method to mitigate such effects and improve the accuracy of BFS measurement. The different expressions of BFS variances presented in previous papers are explained and discussed.

## 1. Introduction

The Brillouin optical fiber distributed sensor is attractive for the measurement of strain and temperature change in fiber under test (FUT), based on the Brillouin frequency shift (BFS), which is a function of strain and temperature. The typical sensitivities were reported as $\partial \nu_{B}/\partial T=1.1\;\mathrm{MHz}/K$ and $\partial \nu_{B}/\partial \varepsilon =48\;\mathrm{kHz}/(\mu \varepsilon )$, where $\mu \varepsilon =10^{-6}$ is the microstrain [1]. Therefore, one of the key issues for the sensor is to extract the peak frequency from the detected signal of the retuned optical wave, which usually contains high-level noise, since the Brillouin scattering is very weak. Obviously, the noise will deteriorate the accuracy of the peak frequency measurement. Some papers have been published discussing the accuracy of BFS. The minimum detectable peak frequency change was given earlier in Ref. [2], expressed as

(1) $$\delta \nu_{B}=\frac{\Delta \nu_{B}}{\sqrt{2}{\left(SNR\right)}^{1/4}}$$

where *SNR* is the signal-to-noise ratio of the detected electrical signal; and $\Delta \nu_{B}$ is the FWHM of the Brillouin scattering spectrum, usually in a Lorentzian waveform. The Brillouin linewidth is typically about 40 MHz, so that the *SNR* for 1 MHz resolution of BFS is required to be 58 dB. This is too high for a conventional sensor to reach. Many technical measures have to be used to suppress the noise.

Ref. [3] presented a detailed analysis about BSF variance based on a quadratic function model, expressed as $y=ax^{2}+bx+c$, where the coefficients a, b, and c meet the requirement of least-square fitting. The peak frequency is estimated as −b/2a, giving the value of BFS. A different formula for the error of the estimated Brillouin peak was deduced as

(2) $$\sigma_{v}=\frac{1}{SNR_{A}}\sqrt{\frac{3d\Delta \nu_{B}}{8\sqrt{2}{\left(1-\eta \right)}^{3/2}}}=\frac{1}{SNR_{A}}\sqrt{\frac{3d\Delta \nu_{B}}{4}}$$

where d is the frequency step of the spectrum, *SNR_A_* is the signal-to-noise ratio of the optical signal amplitude, and *η* is the fraction of peak level, over which a quadratic least-square fitting is carried out. The last expression is for the case of η=1/2. The performances of Brillouin sensors were then characterized based on the model, and consistent with the experimental results.

The difference between Equations (1) and (2) attracts attention. Ref. [4] presented simulated comparisons of the two expressions by using Monte Carlo method with varying frequency step, signal-to-noise ratio, and *Q*-factor. The last parameter is inversely proportional to the FWHM of the Brillouin scattering spectrum, which will change in the case of stimulated Brillouin scattering. It is pointed that the signal-to-noise ratios in Equations (1) and (2) have different definitions with *SNR_A_ = SNR*^1/2^. However, the origin of the difference between Equations (1) and (2) seems not very clear. The dependence of BFS accuracy on the data length used in data processing is not analyzed in detail, which is an important factor in quadratic fitting.

In this work, the expression of BFS uncertainty due to Gaussian noise is deduced strictly in detail based on quadratic fitting with the least-square algorithm, giving new formulas for fitted BFS variance and linewidth varying with data length and the data range’s center, noise levels, frequency step, and others. The analyses are verified by experimental signals from a Brillouin optical domain reflectometer (BOTDR), showing good agreement with each other. The deduction of the new formulas is compared with the model presented in previous publications. The reason for the differences between Formulas (1) and (2) and their applicability are discussed also.

It is shown that the data length and data range’s center deviation relative to the Brillouin peak have a direct impact on the accuracy of the extracted BFS. To mitigate this impact, a method of iterative quadratic fitting is proposed and demonstrated in this paper. It is shown, by way of practical applications to the experimental data, that the method is effective with negligible increase of calculation time.

## 2. Quadratic Fitting Characteristics

The quadratic fitting with $y=ax^{2}+bx+c=a{\left(x+b/2a\right)}^{2}+c-b^{2}/4a$ is used widely in various applications [5,6], and in data processing of noisy Brillouin spectra in distributed fiber sensors, typically in Lorentzian $y_{L}=y_{L0}/[1+4{\left(\nu -\nu_{B}\right)}^{2}/\Delta \nu_{B}^{2}]$, where $y_{L}$ is now the spectrum of the detected Brillouin signal with noise. The accuracy of the extracted BFS depends on data noise and the data range used in fitting, including the length of data and its center, i.e., its symmetry relative to the Brillouin peak $\nu_{B}$. Signal noise will lead to fluctuations of $x_{p}=-b/2a$, and its variance is deduced to be (see Appendix A for detail)

(3) $$\sigma_{\nu }^{2}=\langle {\left(x_{p}-{\bar{x}}_{p}\right)}^{2}\rangle =\frac{\Delta x^{4}d\sigma_{}^{2}}{x_{N}^{5}}[\frac{3x_{N}^{2}}{4}+45{\left(\frac{x_{N}}{2}-x_{p}\right)}^{2}]$$

where d is the frequency spacing of data points, $x_{N}=Nd$ is the length of the spectral range used in fitting. $\Delta x=\sqrt{(b^{2}-4ac)/2a^{2}}$ is the FWHM of the fitted quadratic curve, and $\sigma_{}^{2}=SNR_{A}^{-2}$ is the noise variance. Equation (3) shows that the BFS variance is a quadratic function of $x_{N}/2-x_{p}$. In the case where the fitting data range is centered at the Brillouin peak, the BFS variance reaches its minimum.

(4) $$\sigma_{\nu }^{2}=\frac{3\Delta x^{4}d}{4x_{N}^{3}SNR_{A}^{2}}.$$

**Figure 1:**
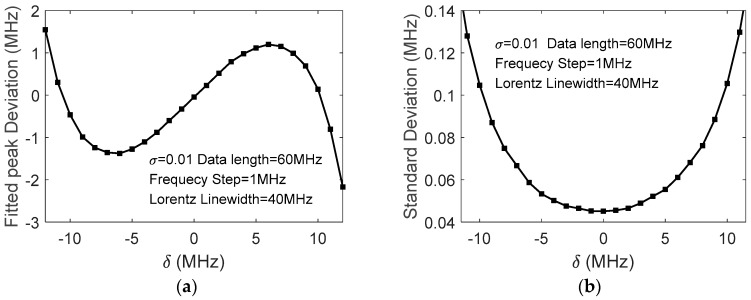
(**a**) Fitted peak frequency deviation (**b**) and standard deviation of fitted peak frequencies vs. data range’s center deviation.

Actually, even if the noise is negligibly small, the quadratic fitted peak may deviate from the Brillouin peak if the center of the data range used in the fitting does not coincide with the latter. Figure 1a shows the deviation of the fitted peak $x_{p}-\nu_{B}$ versus $\delta =x_{N}/2-\nu_{B}$, simulated with d=1 MHz, $x_{N}=60\;\mathrm{MHz}$, and noise level *σ* = 0.01; Figure 1b shows the standard deviations of $x_{p}$ versus *δ*. It is indicated, therefore, that the selections of the data range length and its center in quadratic fitting play important roles in the accuracy of the extracted BFS.

**Figure 2:**
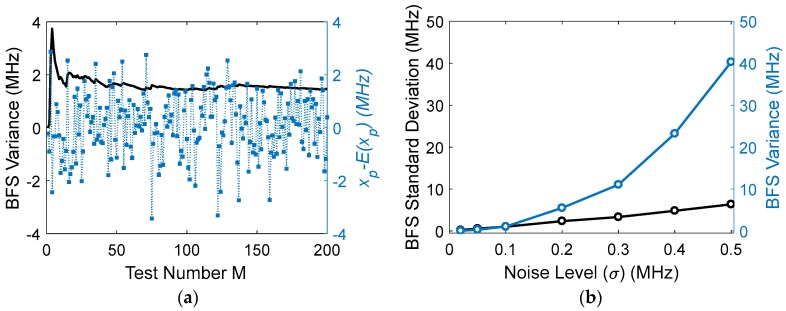
(**a**) Brillouin frequency shift (BFS) variance and its average vs test number, blue dot is BFS of each simulation; (**b**) BFS variances (blue points) and standard deviations (black points) vs. noise level.

The BFS variances obtained by quadratic fitting are simulated with different noise levels. Figure 2a shows an example of BFS variance versus test numbers with σ=0.1, d=1 MHz, $x_{N}=200\;\mathrm{MHz}$, and Lorentzian peak set at the middle of the data range; the blue points are the peak frequencies for different simulation tests, and the black line is the variances obtained by averaging over the test numbers. Obviously, the fitted peaks are randomly distributed due to the noise. Figure 2b gives the variances and standard deviations versus the noise level *σ*. It is seen that the standard deviation is a straight line, showing that the BFS variance is inversely proportional to the square of *SNR_A_*, coincident with Equation (4).

**Figure 3:**
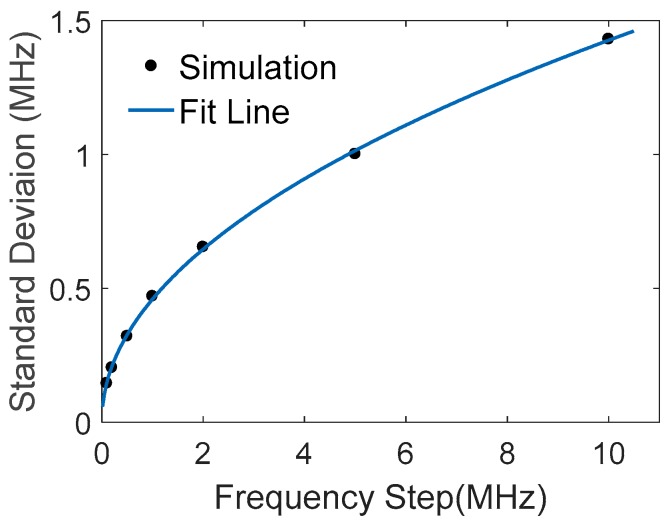
Standard deviation of fitted peak frequencies vs. frequency step.

The relation between BFS variance and frequency step is also studied by simulation with *σ* = 0.1 and $x_{N}=60\;\mathrm{MHz}$. Figure 3 shows that the BFS standard deviation is the square root of the frequency step, coincident with Equation (4); therefore, a smaller step will lead to higher accuracy of the BFS measurement. However, the frequency step d is generally inversely proportional to the probe pulse width Δ*T* of OTDR system, determined by FFT for the optimal spatial resolution. A trade-off has to be taken between BFS accuracy and spatial resolution.

**Figure 4:**
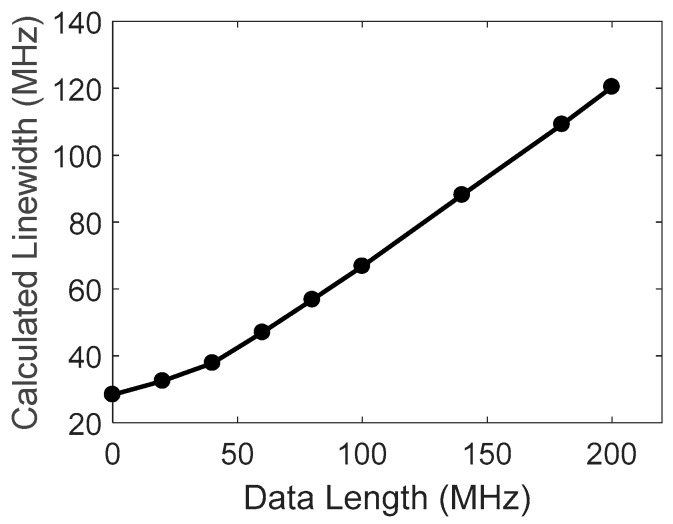
Calculated linewidth of quadratic fitting vs. data number with FWHM of 40 MHz.

The fitted linewidth Δ*x* is an important parameter in Equations (3) and (4). In a previous publication [3], an approximation of $\Delta x∼\Delta \nu_{B}$ was taken. In actuality, it depends on the length of the data used in fitting. The dependence of Δ*x* on $x_{N}$ and $\Delta \nu_{B}$ for the case without noise and without deviation of the data range’s center is deduced to be (see Appendix B for detail)

(5) $$\Delta x={\left[\frac{x_{N}^{2}}{6}\frac{\theta (1+3\rho^{2}/5)-\rho }{\theta (1+\rho^{2}/3)-\rho }\right]}^{1/2}\approx \{\begin{array}{cc} \Delta \nu_{B}(1+9\rho^{2}/40)/\sqrt{2}, & \left(\rho \ll 1\right)\\ px_{N}+q\Delta \nu_{B}, & \left(\rho \gg 1\right) \end{array}$$

where $\rho =x_{N}/\Delta \nu_{B}$; *θ* = tan^−1^*ρ*; and the coefficients of linear approximation in the range of $x_{N}\gg \Delta \nu_{B}$ are calculated to be p=0.548, q=0.465. Figure 4 shows a simulation example, where the frequency step is taken as d=1 MHz, Lorentzian FWHM is 40 MHz, and the noise level is *σ* = 0.02. The coefficients are estimated to be p=0.53 and q=0.35 for this simulation example, in good agreement with the results of Equation (5). The point at $x_{N}=0$ is the theoretical limitation of $\Delta x(x_{N}\to 0)=\Delta \nu_{B}/\sqrt{2}$. Simulations show that this linear relation and the coefficients do not change much for different noise levels.

**Figure 5:**
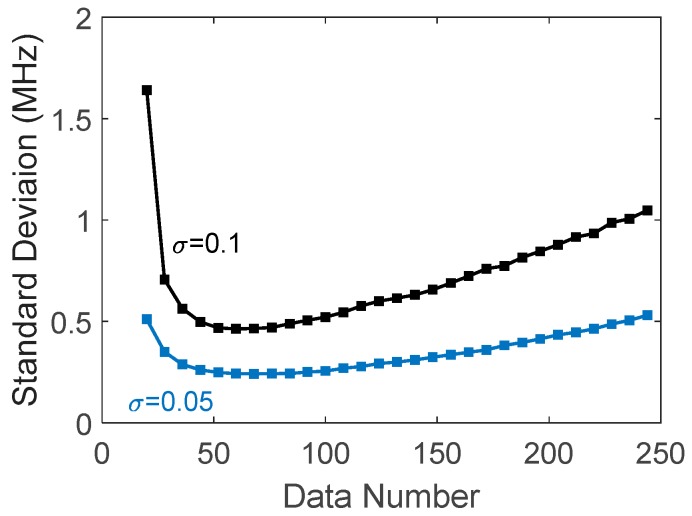
Standard deviation of fitted peak frequencies vs. data number used in fitting.

It is then seen that factor $\Delta x^{4}/x_{N}^{3}$ in Equation (4) will go up towards infinity when $x_{N}$ goes down towards zero, and the rising slope depends on *SNR_A_*. It is reasonable that a decrease in the data number used in fitting will weaken their role in noise reduction, and thus increase the BFS variance, as shown in Figure 5. On the other hand, in the range of $x_{N}\gg \Delta \nu_{B}$, the BFS variance will increase linearly with $x_{N}$; therefore, a minimum is reached at $x_{N}^{\min}=3q\Delta \nu_{B}/p$, and the standard deviation of the peak frequency can then be expressed as

(6) $$\sigma_{\nu }^{}=\frac{8\sqrt{p^{3}q\Delta \nu_{B}d}}{3SNR_{A}}.$$

It is estimated by the analyzed result and the experimental data that $\sqrt{p^{3}q}\sim 0.25$ and 3q/p~2. Figure 5 shows simulated curves of the BFS standard deviation versus data number for two noise levels as examples, where d=1 MHz and $\Delta \nu_{B}=40\;\mathrm{MHz}$ are taken in the simulation, showing the existence of a minimum. 

## 3. Experimental Results and Data Processing

The characteristics analyzed above are found to be in good agreement with our experimental results from a Brillouin optical time domain reflectometer (BOTDR). A narrow linewidth laser with frequency shifted by acousto-optic modulator (AOM) was used as the probe in the experiment; and a Brillouin fiber laser was used as the local oscillator for heterodyne detection, as described in [7,8,9]. A 30 km long sensing fiber (SMF-28) was used in the experiments. The pulse width of the probe was set up as 100 ns; digital signals were given by using a data acquisition card with a sampling rate of 2 GS/s. Then, the Brillouin spectra were obtained by FFT with a frequency step of 1 MHz. The spectra usually contain high-level noise, especially for the signals from longer fiber distances. The frequency difference between Brillouin scattering from fiber and the local oscillation is typically 331 MHz in our BOTDR system.

**Figure 6:**
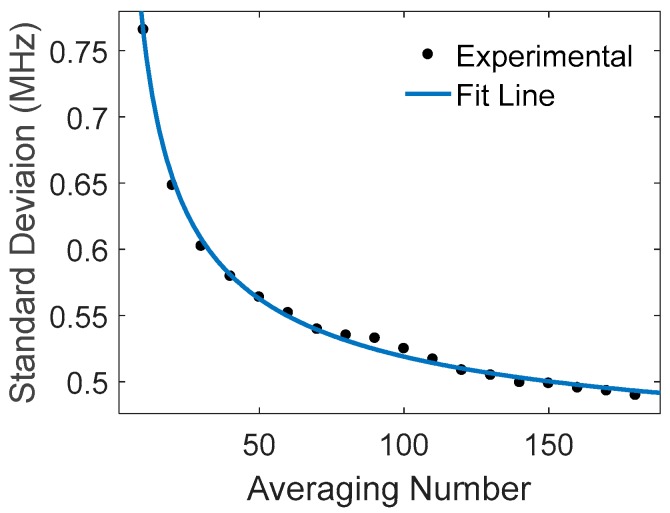
Standard deviation of fitted peak frequencies vs. averaging number.

The *SNR* can be enhanced directly by averaging *M* multiple traces, as $SNR\sim M^{1/2}$. Figure 6 gives the standard deviation of the fitted peak frequency versus the averaging number *M*, showing behavior of $\sigma_{\nu }\sim 1/M^{1/2}$, consistent with Equation (6).

**Figure 7:**
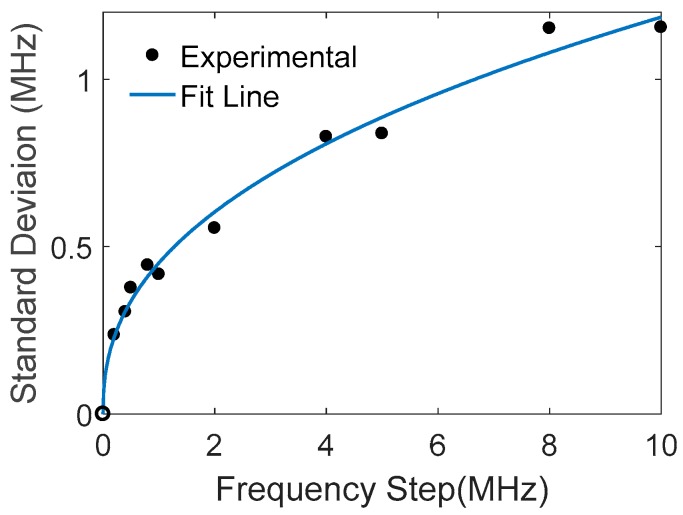
Standard deviation of fitted peak frequencies vs. frequency step.

The frequency step is related to the pulse width of the laser probe, which is set for the optimal spatial resolution. In this study, the spatial resolution is assumed to be not critical; the step can be adjusted in FFT by changing the signal range in the time domain. Figure 7 gives an example of peak frequency standard deviation versus frequency step, where the data are averaged over 10 traces of the BOTDR signal, showing a square root relation as the analysis and simulation described. 

**Figure 8:**
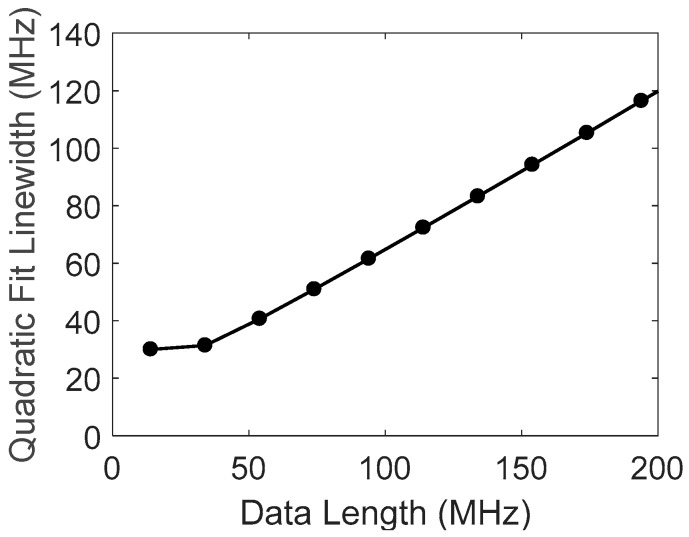
Quadratic fitting linewidths Δ*x* vs. data length.

The FWHM obtained by the quadratic fitting is calculated for different data lengths by using the same spectral signals from the returned wave at a position of 6 km, as shown in Figure 8. The curve is calculated by averaging 10 traces, showing good coincidence with the simulated results of Figure 4. A linear relation appears in the range larger than 50 MHz; the coefficients are obtained as p=0.53, q=0.37; and the FWHM of Brillouin spectrum is estimated to be $\Delta \nu_{B}=32\;\mathrm{MHz}$.

**Figure 9:**
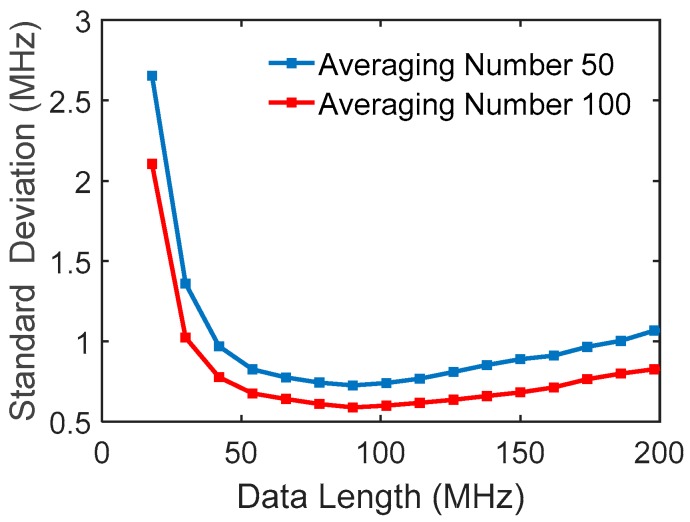
Standard deviation of fitted peak frequencies with data length used in fitting.

The data length used in the fitting is an important parameter which affects the variance of the fitting peak frequency. Figure 9 shows the standard deviation of the fitted peak varied with the data length, where multiple trace signals are used for data processing, and the two curves are for the different averaging numbers. The experimental data were obtained from the returned wave at a fiber distance of 30 km. The data range is centered at the Brillouin peak obtained by averaging 10,000 traces, giving the expected Brillouin peak. They resemble well the simulated curves in Figure 5.

**Figure 10:**
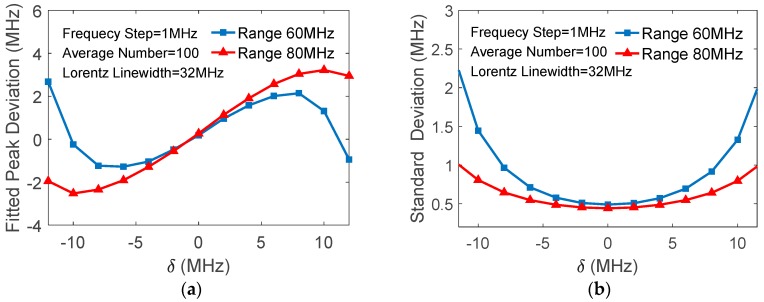
Processing results of experimental data: (**a**) Deviation of fitted peak frequency from Brillouin peak vs data range’s center deviation; (**b**) Standard deviation of fitted peak frequencies vs data range’s center deviation.

The effects of deviation of the data range’s center are verified experimentally. With the same expected Brillouin peak as used in Figure 9, the frequencies and variances of the fitted peaks are calculated for 100 averaged traces, as observed in Figure 10, and are in good agreement with the theoretical analyses.

## 4. Iterative Quadratic Fitting for BFS Measurement

Theoretical and experimental results show that the selection of the data range’s center directly affects the accuracy of the extracted BFS. However, the measured data usually contain high noise and the selected data range’s center in quadratic fitting often deviates from the optimization.

In this work, we propose an iterative quadratic fitting method to reduce the error of the extracted BFS. It is composed of the following steps:

Step 1 Take the frequency at the maximum of the signal amplitude obtained by averaging of *M* traces as the data range’s center $x_{c}$. The averaging number *M* can be adjusted according to the signal noise level.
Step 2 Carry out quadratic fitting for the averaged signal with a selected data range centered at $x_{c}$. A new peak frequency $x_{new}$ is then obtained.
Step 3 Replace $x_{c}$ by $x_{new}$ and repeat Step 2 until the fitted peak converges to a stationary value.

**Figure 11:**
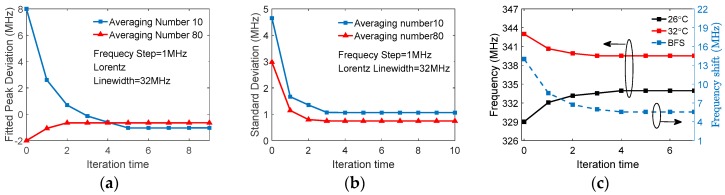
(**a**) Fitted peak deviation vs iteration times and (**b**) standard deviation of fitted peaks vs iteration times; (**c**) The BFS caused by temperature change is extracted by iterative quadratic fitting.

Figure 11a shows the effect of the iterative quadratic fitting method where the experimental data of BOTDR are used; results with two averaging numbers are displayed as examples. For the case of averaging 80 traces at Step 1, the peak frequency convergence is obtained in only two iterations, while five iterations are needed for averaging number of 10. It is noticed that the peak frequency estimated at Step 1 may deviate largely from the converged peak frequency, even positively or negatively, due to the randomness of noise. The two converged results have small differences between each other; this is also due to the noise. To ensure convergence, the initial data center should be selected in the range between two poles with positive slope in Figure 10a.

The effect of the iterative quadratic fitting method can also be examined using the standard deviation of the fitted peak frequencies, as shown in Figure 11b, where the experimental data are divided into multiple groups (e.g., 100–1000) with *M* traces in each, and the fitted peak is then calculated for the multiple groups, giving the multiple results. It is seen that the standard deviations decrease and converge as the iteration time increases. In practical applications, the iterations are stopped when the fitted peak converges under a required accuracy. 

Compared with the conventional fitting method, the iteration method requires fewer averaging numbers for the same accuracy. In our system (Matlab 2016b, 4 CORE I7-4770S, 8 G RAM) the data acquisition and FFT for each trace need 0.03 s, whereas a single quadratic fitting takes only 0.000017 s, which can be negligible. For example, it takes 80 × 0.03 s = 2.4 s and 10 × 0.03 s = 0.3 s, respectively, to get the two curves of Figure 11a. Obviously, the iteration method will improve system speed. The same accuracy of the BFS by the iterative quadratic fitting is demonstrated experimentally in the BOTDR temperature sensor, as shown in Figure 11c. A section of FUT was intentionally heated and the detected signals are averaged over 200 traces with a 1 MHz frequency step. The extracted frequencies converge at 333.9 MHz for a temperature of 26 °C and at 339.5 MHz for 32 °C after three iterations. The obtained BFS of 5.6 MHz is almost the same as that obtained with Lorentzian fitting provided by Matlab (5.5 MHz).

## 5. Discussions

In this work, we proposed a new formula for the minimum detectable peak frequency change. It is in a similar form to that given by Ref. [3], but some different arguments are introduced in the deduction. 

Firstly, the covariance of coefficients a and b are taken into consideration, which we think should not be omitted, as shown in Equation (A5) of the Appendix A. The introduction of the covariance led to Equation (A6), which gives a relation between the variance of the fitted peak frequency and the center of the data range. Secondly, the center of the data range used in fitting relative to the Brillouin peak is an important factor which will lead to an error in the fitted peak frequency. These characteristics have an important influence on the accuracy of the BFS, since the selection of the data range’s center is with some uncertainty due to high noise. To overcome such an effect, we proposed a method of iterative fitting. Thirdly, we noticed that the fitted linewidth of the quadratic curve depends on the data length used in fitting. A linear equation of Δ*x* on $\Delta \nu_{B}$ and $x_{N}$ was deduced theoretically and verified experimentally. This relation is needed in deducing an exact formula for the minimum detectable peak frequency change.

Equation (1) given by Ref. [2] is actually deduced from the quadratic approximation of the Lorentzian curve near the peak frequency, $y\approx y_{0}[1-4{\left(\nu -\nu_{B}\right)}^{2}/\Delta \nu_{B}^{2}]$, where $\Delta \nu_{B}$ is the FWHM of the Lorentzian profile. The detected electrical signal *V* is proportional to y. The peak is determined by its derivative ∂V/∂ν=0, i.e., $\delta V=8V_{0}(\nu -\nu_{B})\delta \nu_{H}/\Delta \nu_{B}^{2}\to 0$, at the peak $\delta V=8V_{0}\delta \nu_{H}^{2}/\Delta \nu_{B}^{2}$, where $\delta \nu_{H}$ is the half width of peak uncertainty. For noisy data, the change of electrical signal depends on its electrical signal-to-noise ratio $\delta V/V_{0}={\left(SNR\right)}^{-2}$. The full width of peak uncertainty is thus obtained as $\delta \nu_{B}=2\delta \nu_{H}=\Delta \nu_{B}/(\sqrt{2}SNR^{1/4})=\Delta \nu_{B}/(\sqrt{2}SNR_{A}^{1/2})$.

In the deduction of this formula, the frequency step d and the data number *N* used for the quadratic fitting are not taken into consideration. The formula may be regarded as having the limitation of d→0 and with Nd finite. In practice, the accuracy of the derivative calculation is surely dependent on d and *N*. The simulation presented by Ref. [4] showed a relation of $\delta \nu_{B}\propto d^{0.05}/SNR^{1/4}$ by using 10,000 Monte Carlo simulations, which, we think, corresponds to the case of N→∞. Therefore, Equation (1) seems to give a qualitative description in ideal cases, but may not be suitable for practical applications. 

It is of note that the analyses in this paper are for Gaussian noise cases. Although the Gaussian noise surely exists, other noise types need to be taken into consideration, such as noise induced by laser sources and devices used in the system [10] and noise due to non-perfect extinction ratio [11]. For a BOTDR with heterodyne detection, the frequency noise and relative intensity noise (RIN) of both the probe laser and the local oscillator are important noise. Besides this, the spectrum may deviate from the strict Lorentzian, such as a convolution of Lorentzian and the pulse shape, as discussed in Ref. [12]. Further studies are undertaken in our group. 

Our study is mainly limited in applications of BOTDR; change of Brillouin linewidth, as in the sensor based on stimulated Brillouin scattering, is not involved. It is believed that the analysis presented in this paper is also useful for BOTDA applications, where the linewidth may be decreased by Brillouin gain.

## 6. Conclusions

Formulas for Brillouin frequency shift extracted from the detected signals by quadratic fitting were deduced strictly in detail. The variances of the fitted BFS and their relations with different noise levels and the data range used in fitting were studied in both simulation and experiments. It is indicated that deviation of data range’s center relative to the Brillouin peak will lead to errors in the BFS measurement; the iterative quadratic fitting method was proposed and demonstrated to improve the fitting accuracy. The formulas presented in previous publications and their contradiction with each other were discussed and explained. The analyses and method are believed beneficial to understanding issues in data processing based on quadratic fitting. Besides BOTDR, this method may be used in other data processing which uses quadratic fitting, such as BOTDA, Raman spectroscopy, etc.

## Supplementary Materials

The following are available online at http://www.mdpi.com/1424-8220/18/2/409/s1, Video S1: A simulation example of fitted peak and its STD vary with the data range.

## Appendix A. Variance of Fitted Peak Frequency

The least square of quadratic fitting requires $f=\sum_{i}{\left[ax_{i}^{2}+bx_{i}+c-y_{i}\right]}^{2}$ minimized for the smallest fitting error. By ∂f/∂a=0, etc., coefficients *a*, *b*, and *c* have to obey the equations

(A1) $$\begin{array}{l} \beta a+\alpha b+Nc=u\\ \gamma a+\beta b+\alpha c=v\\ \kappa a+\gamma b+\beta c=w \end{array}$$

where $\alpha =\sum_{i=1}^{N}x_{i},\;\beta =\sum_{i=1}^{N}x_{i}^{2},\;\gamma =\sum_{i=1}^{N}x_{i}^{3},\;\kappa =\sum_{i=1}^{N}x_{i}^{4}$, $u=\sum_{i=1}^{N}y_{i},\;v=\sum_{i=1}^{N}x_{i}y_{i},\;w=\sum_{i=1}^{N}x_{i}^{2}y_{i}$; $y_{i}$ are the spectral signal amplitudes; $x_{i}=x_{0}+id$ are the frequencies with an equal spacing of *d*; and *N* is the total data number used in the fitting. The start point $x_{0}$ of the fitting can be set to zero without loss of generality. For the case of N≫1, the following approximations are valid: $\alpha \approx N^{2}d/2,\;\beta \approx N^{3}d^{2}/3,\;\gamma \approx N^{4}d^{3}/4,\;\kappa \approx N^{5}d^{4}/5$.

The effect of noise on BFS retrieval can be deduced as follows. The peak frequency from the quadratic curve is at $x_{p}^{}=-b/2a$, and the FWHM of the quadratic curve is $\Delta x=\sqrt{2y_{p}/\left|a\right|}$ with a peak amplitude of $y_{p}=c-b^{2}/4a$. The uncertainty of the peak frequency due to noise is $\delta x_{p}^{}=(-\delta b/b+\delta a/a)x_{p}^{}$. The variance of $\delta x_{p}^{}$ is then written as

(A2) $$\sigma_{\nu }^{2}=x_{p}^{2}[\frac{\langle \delta a^{2}\rangle }{a^{2}}-\frac{2\langle \delta a\delta b\rangle }{ab}+\frac{\langle \delta b^{2}\rangle }{b^{2}}]=\frac{x_{p}^{2}}{a^{2}}[\langle \delta a^{2}\rangle +\frac{\langle \delta a\delta b\rangle }{x_{p}^{}}+\frac{\langle \delta b^{2}\rangle }{4x_{p}^{2}}].$$

The fitting coefficients, *a*, *b*, and *c*, and their fluctuations δa, δb, and δc due to noise, can be obtained by solving Equation (A1), with noisy signals of $y_{i}={\bar{y}}_{i}+\delta y_{i}$, and parameters in the forms $u=\bar{u}+\delta u$, $v=\bar{v}+\delta v$ and $w=\bar{w}+\delta w$. The coefficient deviations δa, δb, and δc are linear functions of δu, δv, and δw. For N≫1,

(A3) $$\begin{array}{l} \delta a\approx 30(N^{2}d^{2}\delta u-6Nd\delta v+6\delta w)/N^{5}d^{4}\\ \delta b\approx -12(3N^{2}d^{2}\delta u-16Nd\delta v+15\delta w)/N^{4}d^{3}. \end{array}$$

The variances of δu, δv, δw and their co-variances can be written as

(A4) $$\begin{array}{l} \langle \delta u^{2}\rangle =\langle \sum_{i=1}^{N}\delta y_{i}^{2}\rangle =N\langle \delta y^{2}\rangle \\ \langle \delta v^{2}\rangle =\langle d^{2}\sum_{i=1}^{N}i^{2}\delta y_{i}^{2}\rangle \approx N^{3}d^{2}\langle \delta y^{2}\rangle /3\\ \langle \delta w^{2}\rangle =\langle d^{4}\sum_{i=1}^{N}i^{4}\delta y_{i}^{2}\rangle \approx N^{5}d^{4}\langle \delta y^{2}\rangle /5\\ \langle \delta u\delta v\rangle =\langle d\sum_{i=1}^{N}i\delta y_{i}^{2}\rangle \approx N^{2}d\langle \delta y^{2}\rangle /2\\ \langle \delta u\delta w\rangle =\langle d^{2}\sum_{i=1}^{N}i^{2}\delta y_{i}^{2}\rangle \approx N^{3}d^{2}\langle \delta y^{2}\rangle /3\\ \langle \delta v\delta w\rangle =\langle d^{3}\sum_{i=1}^{N}i^{3}\delta y_{i}^{2}\rangle \approx N^{4}d^{3}\langle \delta y^{2}\rangle /4 \end{array}$$

where $\langle \delta y^{2}\rangle =\langle \sum_{i=1}^{N}\delta y_{i}^{2}\rangle /N$ is the variance of the optical signal in the frequency domain.

It is indicated by simulations that the approximations of the last expressions of Equation (A4) are valid for large enough *N*. The variance of *a* and *b* can be deduced from Equations (A1) and (A3) as

(A5) $$\begin{array}{l} \langle \delta a^{2}\rangle =180\langle \delta y^{2}\rangle /(N^{5}d^{4})\\ \langle \delta b^{2}\rangle =192\langle \delta y^{2}\rangle /(N^{3}d^{2})\\ \langle \delta a\delta b\rangle =-180\langle \delta y^{2}\rangle /(N^{4}d^{3}) \end{array}.$$

By substituting $\left|a\right|=2y_{p}/\Delta x^{2}$ the variance of the extracted BFS is then obtained as

(A6) $$\sigma_{\nu }^{2}=\frac{3\Delta x^{4}d\sigma_{}^{2}}{x_{N}^{3}}(\frac{15}{x_{N}^{2}}x_{p}^{2}-\frac{15}{x_{N}}x_{p}+4).$$

## Appendix B. Dependence of Fitted Linewidth on Data Length and Brillouin Linewidth

The dependence of the FWHM of fitted quadratic curve on data length and Brillouin linewidth is deduced by using an integral approximation. The least square condition can be written as

(A7) $$f(a,b,c)=\int_{-x_{1}}^{x_{2}}{\left[ax^{2}+bx+c-y(x)\right]}^{2}dx=\min.$$

where $y(x)=1/(1+4x^{2}/\Delta \nu_{B}^{2})$ is the Lorentz-shaped curve without noise and with peak frequency $\nu_{B}=0$ for convenience, $\Delta \nu_{B}$ is the FWHM of the Lorentz-shaped curve; and $-x_{1}∼x_{2}$ is the integral interval. By ∂f/∂a=0, etc., coefficients *a*, *b*, and *c* are thus required to obey the equations

(A8) $$\begin{array}{l} \beta a+\alpha b+x_{N}c=u\\ \gamma a+\beta b+\alpha c=v\\ \kappa a+\gamma b+\beta c=w \end{array}$$

where *α*, *β*, *γ* and $\kappa =\int_{-x_{1}}^{x_{2}}x^{m}dx$, (*m* = 1,2,3,4); u,v, and $w=\int_{-x_{1}}^{x_{2}}yx^{m}dx$, (*m* = 0,1,2). For simplicity, we take the case without data range center deviation, i.e., $x_{1}=x_{2}$; then, the integrals are deduced as $u=\Delta \nu_{B}\theta$, v=0, $w=\Delta \nu_{B}^{2}(x_{N}-\Delta \nu_{B}\theta )/4$, α=γ=0, $\beta =x_{N}^{3}/12$, and $\kappa =x_{N}^{5}/80$, where $\rho =x_{N}/\Delta \nu_{B}$, $\theta =\tan^{-1}\rho$, and $x_{N}=x_{1}+x_{2}$. The fitted coefficient *b* = 0 is also obtained, meaning that the fitted peak coincides with the Brillouin peak. The linewidth of the fitted quadratic curve is then given by

(A9) $$\Delta x^{2}=\frac{2c}{\left|a\right|}=\frac{2(\beta w-\kappa u)}{\left|u\beta -wx_{N}\right|}=\frac{x_{N}^{2}}{6}\frac{\theta (1+3\rho^{2}/5)-\rho }{\theta (1+\rho^{2}/3)-\rho }.$$

By using Tailor expansions of $\theta \approx \rho -\rho^{3}/3+\rho^{5}/5$ for ρ≪1 and θ≈π/2−1/ρ for ρ≫1, the linewidth can be expressed as

(A10) $$\Delta x\approx \{\begin{array}{cc} \Delta \nu_{B}(1+9x_{N}^{2}/40\Delta \nu_{B}^{2})/\sqrt{2} & \left(\rho \ll 1\right)\\ \sqrt{0.3}x_{N}+(8\sqrt{0.3}/3\pi )\Delta \nu_{B} & \left(\rho \gg 1\right) \end{array}.$$

The last expression can be written as $\Delta x\approx 0.548x_{N}+0.465\Delta v_{B}$.

## References

[1] Parker T.R., Farhadiroushan M., Handerek V.A., Rogers A.J. (1997). Temperature and strain dependence of the power level and frequency of spontaneous Brillouin scattering in optical fibers. Opt. Lett..

[2] Horiguchi T., Shimizu K., Kurashima T., Tateda M., Koyamada Y. (1995). Development of a Distributed Sensing Technique Using Brillouin-Scattering. J. Lightwave Technol..

[3] Soto M.A., Thevenaz L. (2013). Modeling and evaluating the performance of Brillouin distributed optical fiber sensors. Opt. Express.

[4] Yu Y.F., Luo L.Q., Li B., Soga K., Yan J.Z. (2016). Frequency Resolution Quantification of Brillouin-Distributed Optical Fiber Sensors. IEEE Photonics Technol. Lett..

[5] Yamamoto M., Sato T., May P.T., Tsuda T., Fukao S., Kato S. (1988). Estimation Error of Spectral Parameters of Mesosphere-Stratosphere-Troposphere Radars Obtained by Least-Squares Fitting Method and Its Lower Bound. Radio Sci..

[6] Richter P. (1995). Estimating Errors in Least-Squares Fitting.

[7] Hao Y.Q., Ye Q., Pan Z.Q., Yang F., Cai H.W., Qu R.H., Zhang Q.Y., Yang Z.M. (2012). Design of Wide-Band Frequency Shift Technology by Using Compact Brillouin Fiber Laser for Brillouin Optical Time Domain Reflectometry Sensing System. IEEE Photonics J..

[8] Hao Y.Q., Ye Q., Pan Z.Q., Cai H.W., Qu R.H., Yang Z.M. (2013). Effects of modulated pulse format on spontaneous Brillouin scattering spectrum and BOTDR sensing system. Opt. Laser Technol..

[9] Cao Y.L., Ye Q., Pan Z.Q., Cai H.W., Qu R.H., Fang Z.J., Zhao H. Mitigation of polarization fading in BOTDR sensors by using optical pulses with orthogonal polarizations. Proceedings of the 23rd International Conference on Optical Fibre Sensors.

[10] Urricelqui J., Soto M.A., Thévenaz L. Sources of noise in Brillouin optical time-domain analyzers. Proceedings of the 24th International Conference on Optical Fiber Sensors (OFS24).

[11] Lu Y., Yao Y., Zhao X., Wang F., Zhang X. (2013). Influence of non-perfect extinction ratio of electro-optic modulator on signal-to-noise ratio of BOTDR. Opt. Commun..

[12] Naruse H., Tateda M. (1999). Trade-off between the spatial and the frequency resolutions in measuring the power spectrum of the Brillouin backscattered light in an optical fiber. Appl. Opt..

